# *CrimsonCalc*: a software tool for pressure determination based on ruby fluorescence spectra

**DOI:** 10.1107/S1600576725007216

**Published:** 2025-08-28

**Authors:** Miha Virant, Matic Lozinšek

**Affiliations:** a Extreme Conditions Chemistry Laboratory (ECCL K2), Jožef Stefan Institute Jamova cesta 39 1000Ljubljana Slovenia; The University of Western Australia, Australia

**Keywords:** high-pressure calculator, ruby fluorescence, diamond anvil cells

## Abstract

*CrimsonCalc* is open-source software for processing ruby fluorescence spectra and calculating pressure from the shift of the *R*_1_ peak. It supports processing and plotting of an individual spectrum as well as batch processing of spectra from data in text or Bruker *OPUS* binary format. Core features include baseline correction, pseudo-Voigt peak fitting, automated detection of detector saturation, and pressure calculation using user-defined or automatically detected reference spectra. The software provides batch output in both human-readable and machine-readable formats. With an intuitive graphical user interface, *CrimsonCalc* is designed as a high-pressure research aid and includes additional tools such as a diamond Raman edge pressure calculator, an interferometry-based gasket thickness calculator and an estimator for the upper pressure limit based on diamond anvil culet diameter. The program can be easily adopted in high-pressure research laboratories to facilitate pressure readings directly from simple text data, and to generate the images and result files that enable straightforward report preparation.

## Abstract 2

Software optimized to fit ruby fluorescence spectra, automatically extract the peak positions and calculate the pressure according to the Ruby2020 gauge is presented.

## 1. Introduction

High-pressure crystallography has emerged as a powerful tool for probing the structural behaviour of materials under extreme conditions. Subjecting samples to pressures reaching hundreds of gigapascals (1 GPa = 10 000 bar), comparable to those found deep within planetary interiors, enables the discovery of new phases and phase transitions, as well as the investigation of fundamental changes in bonding and electronic structure. These insights are essential not only for advancing chemistry, condensed matter physics and materials science but also for understanding geological, geophysical and planetary processes.

The diamond anvil cell (DAC) is the most widely used device for generating static high pressures in the laboratory. In a DAC, the sample is compressed between the culets of two opposing diamond anvils. Owing to the broad transparency of diamond across the electromagnetic spectrum, structural investigations of a sample under pressure in a DAC can be carried out using a range of spectroscopic and diffraction techniques, including X-ray diffraction (XRD) and Raman spectroscopy. XRD, in particular, plays a central role in precise structural characterization and has enabled the discovery of numerous exotic high-pressure phases (Katrusiak, 2008 ▸; Shen & Mao, 2017 ▸; Dubrovinskaia & Dubrovinsky, 2018 ▸).

Accurate pressure determination is a cornerstone of high-pressure studies and is critical for correlating structural changes with thermodynamic parameters. Among the various pressure calibration methods available, the ruby fluorescence technique (Forman *et al.*, 1972 ▸) has become standard due to its robustness, reproducibility and relative ease of use. Ruby (Cr^3+^-doped Al_2_O_3_) exhibits sharp fluorescence lines (*R*_1_ and *R*_2_) when excited by laser light, with the *R*_1_ line shifting to longer wavelengths as pressure increases (Syassen, 2008 ▸). Since its introduction, multiple calibrations of the ruby fluorescence scale have been developed and reported in the literature (Mao *et al.*, 1986 ▸; Chijioke *et al.*, 2005 ▸; Holzapfel, 2005 ▸; Silvera *et al.*, 2007 ▸; Dewaele *et al.*, 2008 ▸; Syassen, 2008 ▸). In 2020, the refined calibration IPPS-Ruby2020 was introduced (Shen *et al.*, 2020 ▸), which is based on extensive experimental data and cross-validation with other primary pressure standards. The Ruby2020 pressure gauge, valid up to 150 GPa, is given by
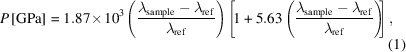
where λ_ref_ and λ_sample_ are the *R*_1_ fluorescence wavelengths at ambient and high pressure, respectively.

Accurate extraction of the fluorescence peak positions from experimental spectra is crucial yet not always trivial. *CrimsonCalc* is a Python-based open-source program with a graphical user interface (GUI) tailored to this task. It streamlines the extraction and processing of the ruby fluorescence data and enables the application of various baseline correction methods, fitting of the resulting spectra with pseudo-Voigt functions and calculation of pressure. Additional tools include a diamond Raman edge pressure calculator, an interferometry-based gasket thickness calculator and an estimator for the upper pressure limit based on diamond anvil culet diameter.

## 2. Program description

### 2.1. Architecture

*CrimsonCalc* is written in Python and integrates several open-source libraries to streamline spectral data analysis and visualization. The GUI was built using the *Tkinter* package (https://wiki.python.org/moin/TkInter), providing a lightweight and accessible front end. Spectral data are processed using the *NumPy* (Harris *et al.*, 2020 ▸), *SciPy* (Virtanen *et al.*, 2020 ▸), *pybaselines* (Erb, 2025 ▸) and *LMFIT* (Newville *et al.*, 2025 ▸) packages. The spectra and calculated parameters are visualized using the *Matplotlib* package (Hunter, 2007 ▸) and can be exported as publication-quality images and tab-delimited text files for further analysis or reporting.

**Figure 1:**
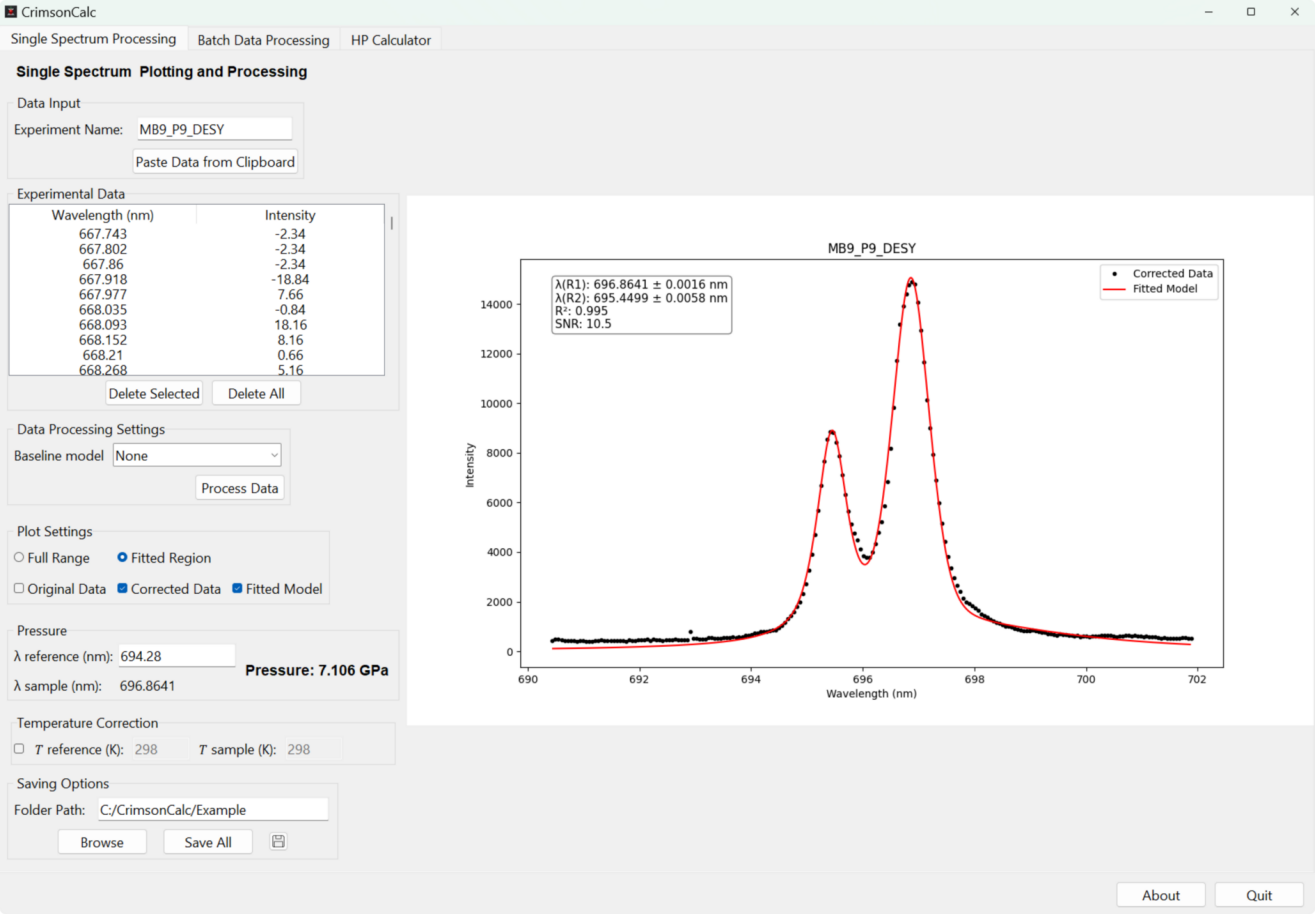
The GUI and the ‘Single Spectrum Processing’ tab with the processed experimental data.

The GUI features a simple three-tab layout, with the ‘Single Spectrum Processing’ tab primarily focused on analysis and plotting of a single spectrum imported via a text-based clipboard (Fig. 1 ▸), the ‘Batch Data Processing’ tab supporting batch processing of ruby fluorescence spectral files and automating the complete workflow from the experimental data input to the final pressure values, and the ‘HP Calculator’ tab serving as a collection of useful tools for high-pressure research. All tabs feature intuitive input fields and clearly labelled buttons to facilitate user interaction.

### 2.2. The *R*_1_ peak position extraction algorithm

The algorithm used to determine the fitted model function and the peak position of the *R*_1_ ruby fluorescence line follows the same procedure in both the ‘Single Spectrum Processing’ and the ‘Batch Data Processing’ tabs. The input data, *i.e.* wavelength and intensity, are first subjected to baseline correction. The baseline-corrected spectrum is then analysed using the find_peaks function from the scipy.signal module (Virtanen *et al.*, 2020 ▸) to obtain preliminary estimates of the *R*_1_ and *R*_2_ peak positions. These initial values serve as starting parameters for the LMFIT.models module (Newville *et al.*, 2025 ▸), which fits the data using a composite model of pseudo-Voigt functions. The fitting process employs an intensity-weighted scheme and the leastsq optimization algorithm from *SciPy*. Final model parameters are extracted and saved in a user-friendly tab-delimited text format and visualized using the *Matplotlib* package (Hunter, 2007 ▸), enabling immediate inspection of the fitted region and overall fit quality.

The choice of the pseudo-Voigt function stems from its suitability for modelling spectral peaks influenced by both Lorentzian and Gaussian broadening (Wertheim *et al.*, 1974 ▸). As a linear combination of Gaussian and Lorentzian components, the pseudo-Voigt function provides a flexible and computationally efficient approximation to the more complex Voigt profile. This makes it especially well suited for analysing Raman and ruby fluorescence spectra, where accurate resolution of peak shifts and shapes is crucial, particularly in applications such as pressure calibration in DAC experiments under extreme pressure–temperature conditions.

At higher pressures, under non-hydrostatic conditions (Takemura, 2021 ▸), a shoulder feature has been observed on the high-wavelength side of the *R*_1_ peak (Motaln *et al.*, 2025 ▸), which can reduce the accuracy of the peak fitting and affect the determination of the *R*_1_ centre position. To address this, the signal-to-noise ratio (SNR) is estimated as the ratio of the mean intensity to the standard deviation of the residuals between the experimental data and the fitted model. If the SNR estimate falls below a threshold value of 10, an additional pseudo-Voigt peak is introduced to the model to fit the shoulder on the *R*_1_ peak. The final position of the *R*_1_ centre obtained from the fitted model is then used to calculate pressure according to the Ruby2020 gauge [equation (1)]. The quantitative measures for the quality of the final fitted model, namely the *R*^2^ and SNR estimate, are provided on the individual plots as well as in the final result file.

### 2.3. ‘Single Spectrum Processing’ tab

In the ‘Single Spectrum Processing’ tab (Fig. 1 ▸), the ‘Data Input’ frame is used to set the experiment name and import the spectral data either via the clipboard by pressing the corresponding button or by drag-and-dropping the corresponding file into the *CrimsonCalc* window.

The imported text is automatically filtered to retain only lines containing two numerical values (whitespace- or comma-delimited), which are interpreted as wavelength and intensity pairs. These values are stored in a table and simultaneously plotted in the plot window on the right for immediate inspection. The user can manually inspect the tabulated data in the ‘Experimental Data’ frame and remove selected parts of the data using the ‘Delete Selected’ button. In the next step, the baseline correction model is selected via a dropdown menu in the ‘Data Processing Settings’ frame. Currently, in addition to no baseline correction (option ‘None’), there are three models from the *pybaseline* package (Erb, 2025 ▸) implemented, namely rubberband, BEADS (baseline estimation and denoising with sparsity) (Ning *et al.*, 2014 ▸) and FABc (fully automatic baseline correction) (Carlos Cobas *et al.*, 2006 ▸). Baseline correction is often crucial, as it enhances the reliability of the subsequent peak fitting procedure, ideally without introducing significant distortion to the spectral features (Schulze *et al.*, 2005 ▸).

Once a model is selected, pressing the ‘Process Data’ button initiates baseline correction followed by peak fitting using the algorithm described in the previous section. The resulting position of the *R*_1_ fluorescence peak is displayed in the ‘Pressure’ frame, along with the corresponding pressure value calculated from the user-input reference wavelength value (λ_ref_) and the Ruby2020 gauge [equation (1)]. Since the ruby fluorescence measurements are sensitive to temperature changes (Datchi *et al.*, 2007 ▸; Syassen, 2008 ▸), a ‘Temperature Correction’ frame is provided, allowing users to enter the temperatures of the reference and sample measurements and obtain the temperature-corrected pressure in the ‘Pressure’ frame above.

The ‘Plot Settings’ provide interactive controls for toggling between the full spectral range and the enlarged view of only the fitted region. The visualization of the original data, the baseline-corrected data and the fitted model is selected via checkboxes. Processed results can be saved to a user-specified directory using the ‘Browse’ button. Clicking the ‘Save All’ button generates a set of files using the ‘Experiment Name’ as the base for the file names. In this way the original data, the corrected spectrum and the fitted model parameters are exported as three tab-delimited text files. In addition, the currently displayed plot in both raster (.png) and vector (.svg) graphics formats is exported, ensuring compatibility with a wide range of analysis and publication tools. Clicking the floppy-disk save button appends the latest results to the default CrimsonCalc_results.txt file located on the desktop. Each entry includes a timestamp, a brief description of the export (*e.g.* Single Spectrum Processing) and the pressure value along with the sample and reference wavelengths (Fig. S1 of the supporting information).

### 2.4. ‘Batch Data Processing’ tab

**Figure 2:**
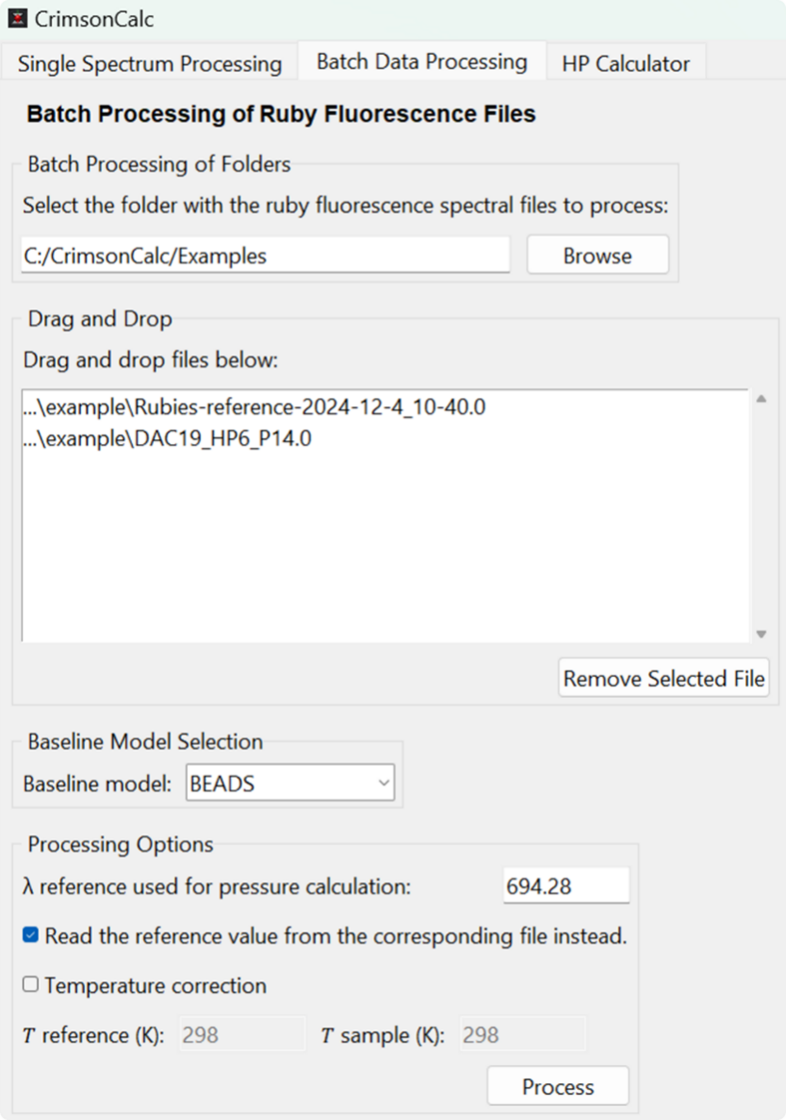
The ‘Batch Data Processing’ tab, which enables batch analysis of spectral files through folder selection or drag-and-drop file input.

The second tab (Fig. 2 ▸) is dedicated to batch processing of ruby fluorescence spectra. Users can either select an entire folder for processing or drag-and-drop individual files into the designated input area. The deduplication ensures that each file is processed only once. The batch processing operates on text-based files each containing a single spectrum (with the .txt extension) and on Bruker *OPUS* binary files (with the .number extension) which may contain multiple spectra per file.

In batch processing, files are categorized according to whether the filename contains the keyword ‘reference’. Files identified as references are used to determine the reference wavelength (λ_ref_), *i.e.* the *R*_1_ fluorescence line collected under ambient conditions, and are processed first. Currently, only the reference value from the first processed reference file in a batch can be used for pressure calculations in the case that multiple reference files are listed for processing. Files without the ‘reference’ keyword in the filename are treated as pressure measurement data. In this case, in addition to the *R*_1_ fluorescence line extraction, pressure is calculated using the selected λ_ref_ value. When the ‘Temperature Correction’ option is enabled, the final pressure value is adjusted (Datchi *et al.*, 2007 ▸) on the basis of the provided temperatures for the reference and sample measurements (only one set of temperature values can be used at a time).

The batch-processing procedure is started by clicking the ‘Process’ button. Each spectrum is subjected to the selected baseline correction method, followed by pseudo-Voigt fitting of the *R*_1_ and *R*_2_ fluorescence peaks as described before. The resulting parameters are saved alongside the input files in their corresponding original directories. Outputs include a result file with the fitted model and calculated pressures, baseline-corrected spectra, and graphical plots in both raster (.png) and vector (.svg) formats. The processing results for all spectra from the text files are compiled into a single file named Ruby_Report.txt, saved in the same directory as the first .txt file in the processed batch. For Bruker *OPUS* files containing multiple spectra (see Section 3.2, *Example 2: a reference and a pressure file containing multiple spectra*[Sec sec3.2]), batch processing outputs not only the results of fitting for each individual spectrum but also the average *R*_1_ peak position for reference files or the average calculated pressure for pressure measurement files. In addition, Bruker *OPUS* files can contain information about detector saturation. These data are used to verify the spectral quality, and only ruby fluorescence spectra that fall below the saturation threshold are processed. Spectra that exceed this threshold are skipped, and their filenames are recorded in a file named _overflow.txt, which is created alongside the _result.txt file.

### 2.5. ‘HP Calculator’ tab

**Figure 3:**
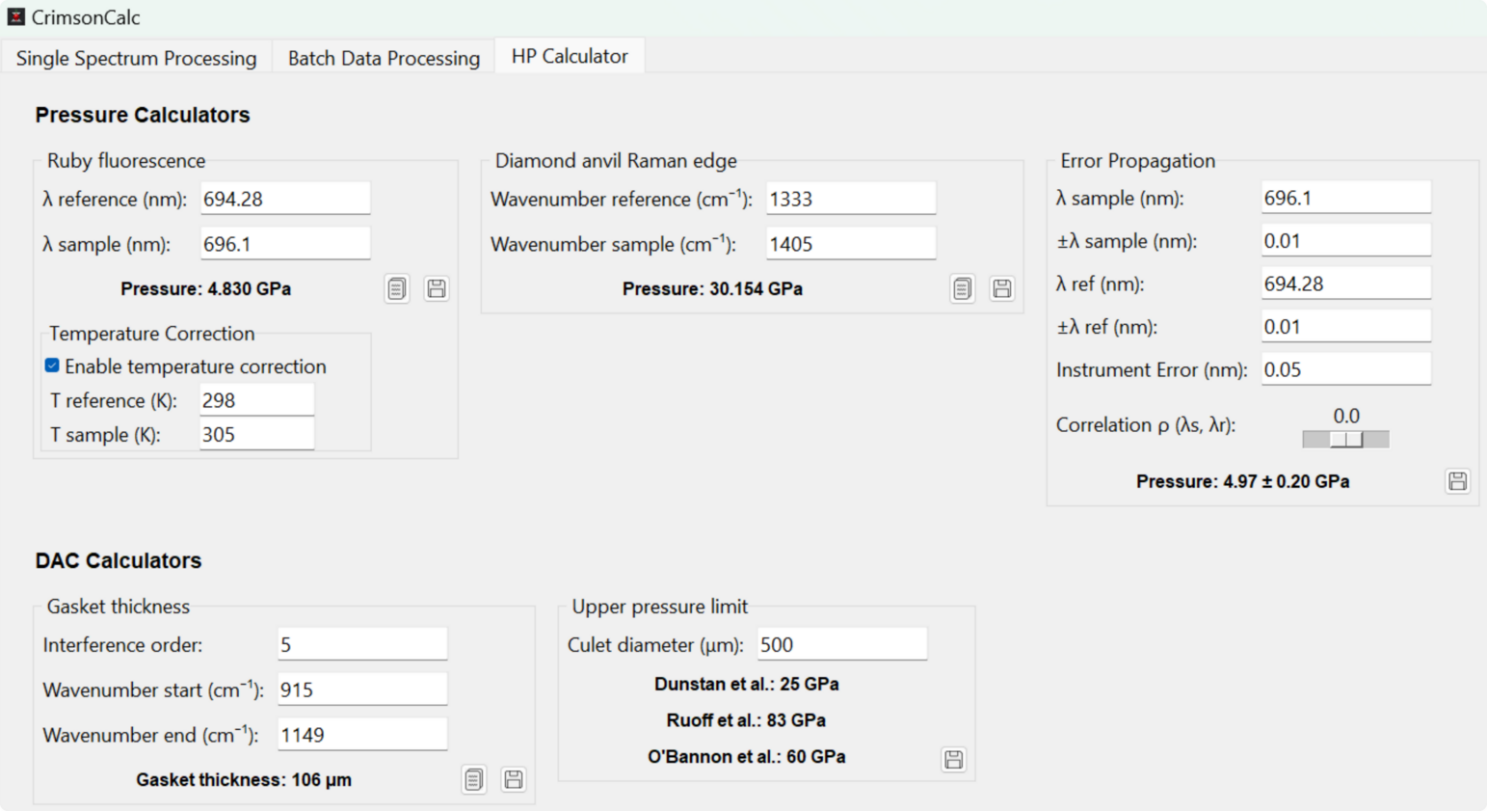
The ‘HP Calculator’ tab featuring five tools useful for high-pressure experiments.

The third tab, titled ‘HP Calculator’ (Fig. 3 ▸), offers a suite of tools useful for high-pressure research. The ‘Pressure Calculators’ replicate the functionality of the widely utilized web-based tools (Kantor, 2025 ▸), providing an offline alternative. This Python-based implementation currently supports two pressure-determination methods, namely the ruby fluorescence based on the Ruby2020 gauge (Shen *et al.*, 2020 ▸) with temperature correction option (Datchi *et al.*, 2007 ▸), also used in the first two tabs, and the Raman diamond edge method (Eremets *et al.*, 2023 ▸). A third tool in the ‘Pressure Calculators’ section estimates the uncertainty associated with the Ruby2020 gauge. It accounts for uncertainties arising from the calibration curve, instrumental error, fitted peak positions, and correlations between the errors in sample and reference measurements. In contrast to the first two tabs, which are dedicated to processing experimental spectral data, this module enables pressure calculations from user-input values. This approach enhances the flexibility of the software and broadens its applicability beyond direct spectral analysis.

Additional tools in this tab include a gasket thickness calculator based on the optical interferometry method (Kim *et al.*, 2021 ▸) and a pressure limit estimator, which provides upper-bound pressure estimates for diamond anvils of specified culet diameters in micrometres, *d*, derived from three published models: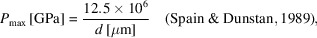 ▸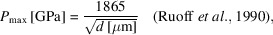 ▸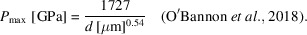 ▸

Note that culet size is not the sole determining factor in achievable pressure, and these estimates should be interpreted with caution.

The calculators in the ‘HP Calculator’ tab also include buttons that allow users to either copy the calculated value to the clipboard (notes icon) or append the results (floppy-disk icon) to the default CrimsonCalc_results.txt file on the desktop. Appended entries include the timestamp, a brief description, the calculated result and the corresponding input values (Fig. S1).

## 3. Usage examples

### 3.1. Example 1: single spectrum in plain-text format

This utility is aimed at users who collect ruby fluorescence data from various spectrometers, and it can export the raw data in whitespace- or comma-delimited plain-text format. It enables rapid pressure calculation through spectral analysis using the ‘Single Spectrum Processing’ tab. An example from the offline ruby system at the Extreme Conditions Beamline P02.2 (Liermann *et al.*, 2015 ▸) at PETRA III, DESY, is presented in Fig. 1 ▸. The displayed spectrum corresponds to a ruby at 7.1 GPa in a DAC, with nitrogen used as the pressure-transmitting medium (PTM).

The general workflow starts with copying the text data either from a file or from the instrument software into the ‘Experimental Data’ window of the ‘Single Spectrum Processing’ tab by pressing the ‘Paste Data from Clipboard’ button. Clicking the ‘Process Data’ button performs the fitting. The best results are often achieved using the BEADS method for baseline correction. However, in some cases the default BEADS background method may fail to model the baseline accurately. When this occurs, alternative models can be tested by selecting a different baseline algorithm, re-processing the spectrum and inspecting the outcome using the ‘Plot Settings’ controls.

### 3.2. Example 2: a reference and a pressure file containing multiple spectra

**Figure 4:**
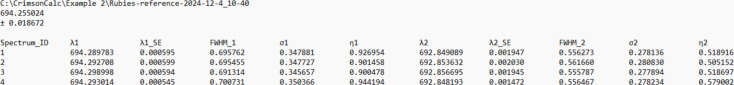
The first few lines of the result file from processing the Bruker *OPUS* reference file with multiple measurements (10 rubies, each ruby was measured on 9 spots).

A folder containing the files Rubies-reference-2024-12-4_10-40.0 and DAC19_HP6_P14.0 measured on a Bruker Senterra II was processed using the ‘Batch Data Processing’ tab. The BEADS method was selected as the baseline model, and the option to read the reference value from the corresponding file was enabled (Fig. 2 ▸). As a result, the reference file was processed first. This file contained 90 spectra acquired from 10 ruby spheres, each measured 9 times. The average reference wavelength and the standard deviation are written in the second and third lines of the output file, respectively (Fig. 4 ▸). Below the average result, tab-delimited fitted results for individual spectra are listed. The first column, Spectrum_ID, refers to either the file name or the spectrum number in cases where a file contains multiple spectra. The subsequent columns contain the fitted parameters for the *R*_1_ and *R*_2_ peaks and, if detected, also for the *R*_3_ shoulder peak. The values λ_*i*_, λ_*i*__SE, FWHM_*i*_, σ_*i*_ and η_*i*_ represent the wavelength of the *R*_*i*_ peak maximum, the estimated standard error from the fitted model, the full width at half-maximum, the standard deviation of the underlying Gaussian component, and the mixing fraction between Lorentzian and Gaussian components, respectively.

**Figure 5:**
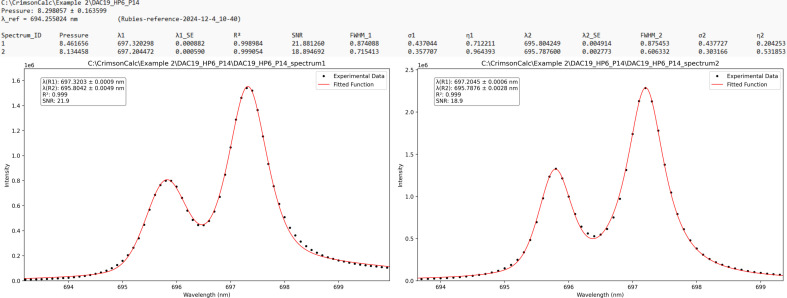
Results from processing the Bruker *OPUS* file from the measurement of two rubies under pressure (Motaln *et al.*, 2025 ▸). The result file report (top) and the two extracted spectra with fitted models (bottom).

The average reference value of 694.255 nm calculated from spectra in the reference file is then used for subsequent pressure calculations with the Ruby2020 pressure gauge [equation (1)]. The file DAC19_HP6_P14.0 contained measurements from two rubies placed inside the DAC. The calculated pressures were 8.46 and 8.13 GPa, respectively. The difference between the two values indicates non-hydrostatic conditions in the sample chamber, consistent with the use of Fomblin Z25 as the PTM (Motaln *et al.*, 2025 ▸). The fitting results were satisfactory without requiring the inclusion of a third peak, and visual inspection confirmed the absence of a shoulder feature (Fig. 5 ▸).

### 3.3. Example 3: a shoulder at non-hydrostatic conditions

**Figure 6:**
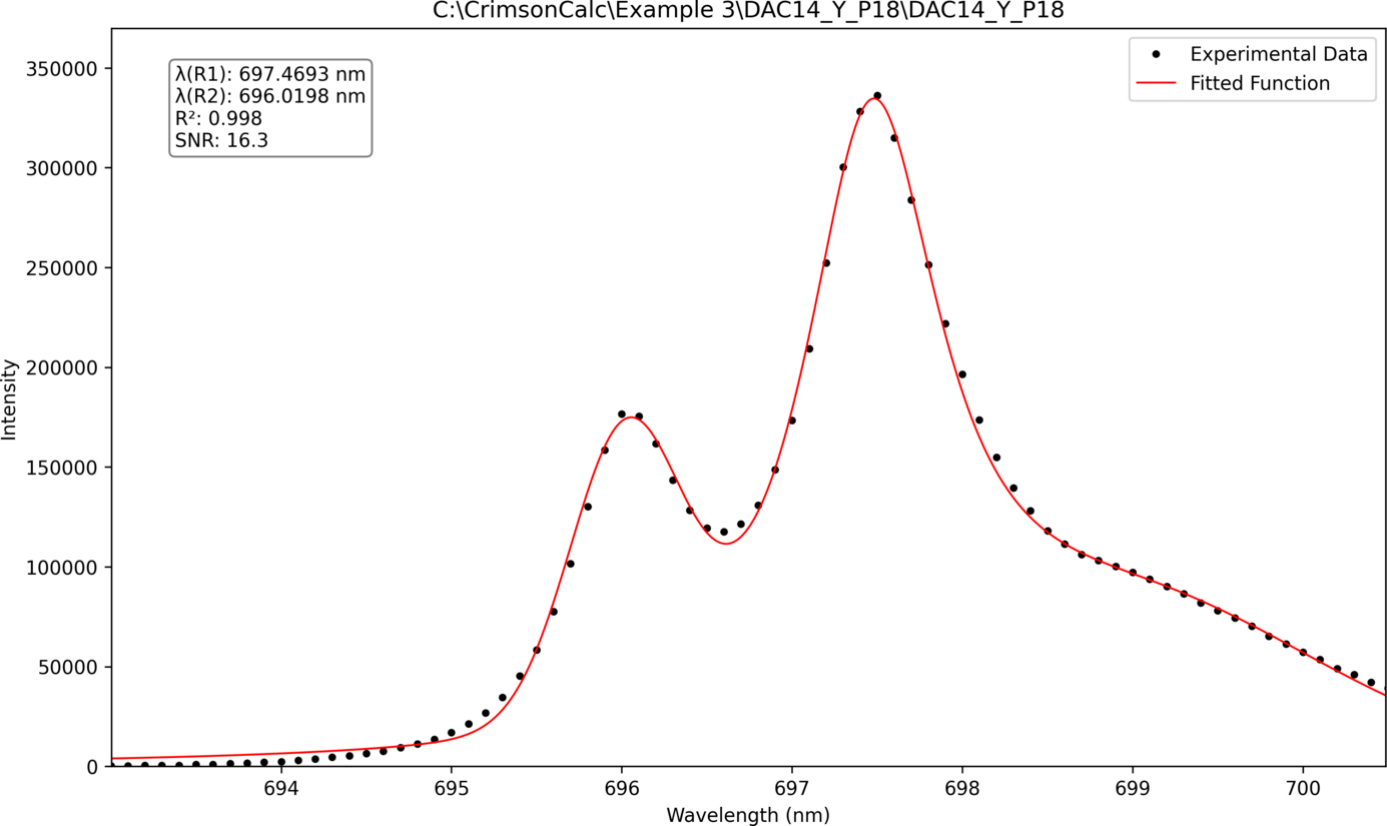
A fluorescence spectrum of a ruby in Fomblin Y oil at 8.8 GPa (Motaln *et al.*, 2025 ▸) with an evident shoulder peak.

As previously reported (Motaln *et al.*, 2025 ▸), ruby fluorescence spectra may exhibit a shoulder on the higher-wavelength side of the *R*_1_ peak when measured under non-hydrostatic conditions. This behaviour was successfully modelled using the *R*_1_ peak position extraction algorithm (FABc baseline model), which detected and fitted the shoulder at 699.082 nm in the presented dataset (Fig. 6 ▸). Although the additional peak was correctly identified and fitted, the uncertainty values associated with the least-squares optimization procedure could not be computed. Consequently, these uncertainties are not reported in the output.

## 4. Availability and distribution

*CrimsonCalc* is distributed under the GNU General Public License, version 3. Documentation and source code (in the Python language) as well as Windows binaries are available at the Extreme Conditions Chemistry Laboratory website (https://eccl.ijs.si/) under the ‘Software’ section. This software was developed with assistance from ChatGPT (OpenAI, 2025 ▸), which provided valuable guidelines and solutions in the code-writing process.

## 5. Outlook

While *CrimsonCalc* currently supports processing of spectral data in plain-text format and Bruker *OPUS* binary format, expanding compatibility to include proprietary binary formats used by different instrument manufacturers would significantly broaden its potential user base. Future development will likely focus on incorporating libraries or modules capable of reading and interpreting spectral data from a wider range of spectroscopy systems. Planned improvements also involve the processing of diamond Raman edge measurements for pressure determination as well as interference spectrum processing for gasket thickness calculations.

## 6. Conclusions

*CrimsonCalc* has been developed as a dedicated software solution for extracting pressure values from ruby fluorescence spectra, enabling automated and efficient processing of experimental data. It features a robust and optimized peak-fitting algorithm that minimizes the need for user intervention, promoting consistency and reproducibility in high-pressure measurements. In addition to streamlined data analysis, the software generates supporting materials suitable for documentation and reporting. By integrating several practical tools for high-pressure research into a single program, *CrimsonCalc* enhances usability and supports a more efficient experimental workflow.

## Supplementary Material

Supporting information file. DOI: 10.1107/S1600576725007216/oc5047sup1.pdf

## Data Availability

The data supporting the findings of this study are available within the article and its supporting information. The *CrimsonCalc* software, including source code (in Python), documentation and precompiled Windows binaries, is freely accessible at the Extreme Conditions Chemistry Laboratory website under the ‘Software’ section: https://eccl.ijs.si/.
