# Supplementary material for: CrimsonCalc: a software tool for pressure determination based on ruby fluorescence spectra
Source: J Appl Crystallogr. 2025 Aug 28;58(Pt 5):1827–33. doi: 10.1107/S1600576725007216 (PMC12502879; doi:10.1107/S1600576725007216)
Supplement: Supplementary file 1 [file j-58-01827-sup1.pdf]

JOURNAL OF APPLIED CRYSTALLOGRAPHY

Volume 58 (2025)

Supporting information for article:

CrimsonCalc: a software tool for pressure determination based on ruby fluorescence spectra

Miha Virant and Matic Lozinšek

S1. CrimsonCalc_results.txt file

Clicking on the floppy-disk button creates or updates a default file for quick saving of the results on the Desktop, titled `CrimsonCalc_results.txt`. As described above, each click on the floppy-disk button appends the corresponding results to the file together with the timestamp, short description and input values (Figure S1).

```
2025-06-01 07:00:00    Single Spectrum Processing:    DAC6_P8 3.862   695.6976        694.28
2025-06-01_08:00:00    Ruby pressure:   4.974   694.28  696.1
2025-06-01 09:00:00    Diamond Edge Pressure:   30.154  1333    1405
2025-06-01 10:00:00    Error Propagation calculation:  4.97 ± 0.20     696.1   0.01    694.28  0.01    0.05    0.0
2025-06-01 11:00:00    Gasket Thickness:       106     5       915     1149
2025-06-01 12:00:00    Upper Pressure Limit:   500     25      83      60
```

Figure S1 An example of the `CrimsonCalc_results.txt` file.

S2. Processing of ruby fluorescence spectrum files

S2.1. Measurement performed on Horiba Jobin-Yvon LabRAM HR spectrometer

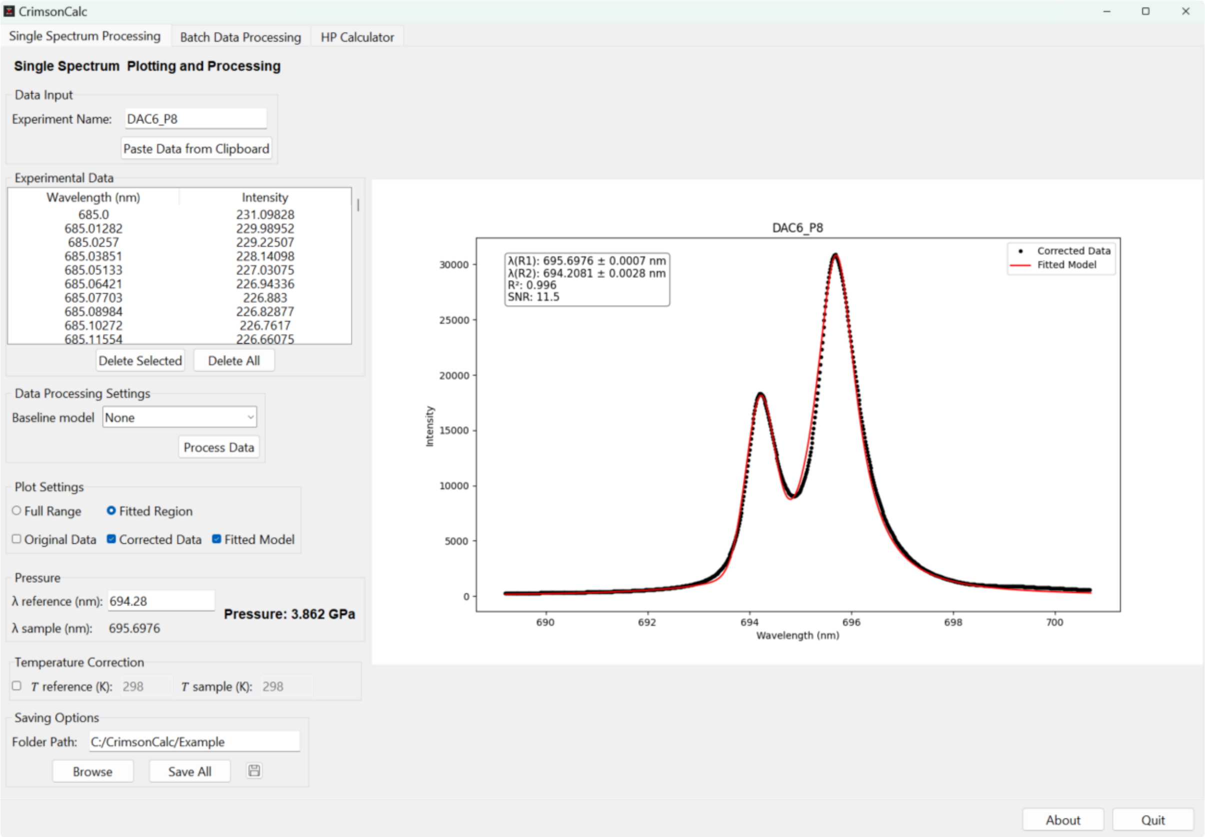

Figure S2 Processing of the ruby fluorescence spectrum measured at 3.9 GPa with the Horiba Jobin-Yvon LabRAM HR spectrometer in ‘Single Spectrum Processing’ tab.

The ruby fluorescence spectrum recorded using the Horiba Jobin-Yvon LabRAM HR spectrometer was processed by copying the plain text data file into the Experimental Data window of the ‘Single Spectrum Processing’ tab (Figure S2). The spectral data was processed using no baseline correction

model and the best fit is composed of two pseudo-Voigt functions with peak centres at 695.70 nm and 694.21 nm. The position of the R_1 peak at 695.70 nm corresponds to the pressure of 3.9 GPa (the reference wavelength of 694.28 nm was used).

S2.2. Measurement performed on Bruker Senterra II spectrometer

The ruby fluorescence spectrum obtained on the Bruker Senterra II spectrometer saved as a plain text file was pasted in the 'Single Spectrum Processing' tab (Figure S3). The spectral data was processed using the FABc baseline correction model and the best fit is composed of two pseudo-Voigt functions with peak centres at 696.40 nm and 694.97 nm. The position of the R_1 peak at 696.40 nm corresponds to the pressure of 5.8 GPa (the reference wavelength of 694.28 nm was used).

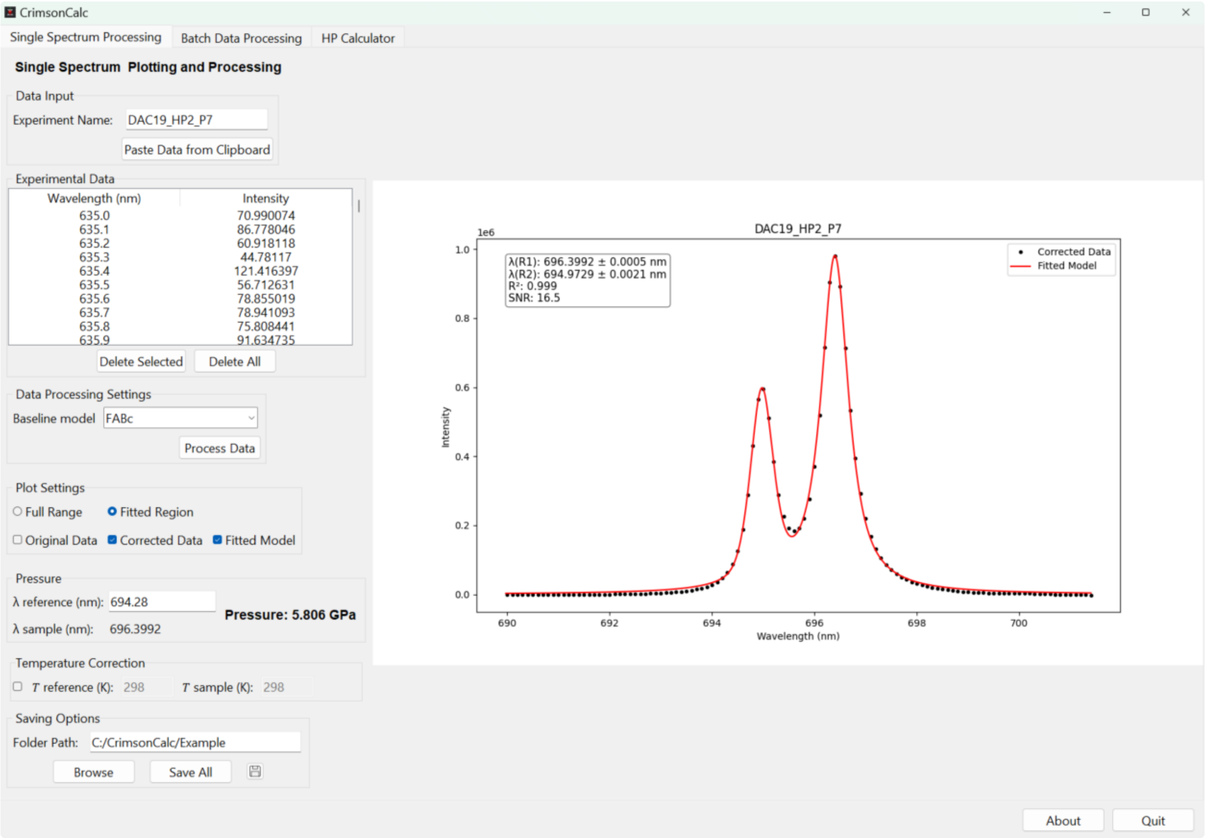

Figure S3 Processing of the ruby fluorescence spectrum measured at 5.8 GPa with the Bruker Senterra II system in 'Single Spectrum Processing' tab.

S3. Error propagation calculation

The 'Error Propagation' calculator in 'HP Calculator' tab is used to calculate the error propagation in case of the Ruby2020 pressure gauge equation (1) rewritten in a form:

$$P[\mathrm{GPa}] = A \times 10^3 \left(\frac{\Delta\lambda}{\lambda_{\mathrm{ref}}}\right)\left[1 + B\left(\frac{\Delta\lambda}{\lambda_{\mathrm{ref}}}\right)\right] \qquad (2)$$

where A = 1.87 GPa, B = 5.63, and $\Delta\lambda = \lambda_{\mathrm{sample}} - \lambda_{\mathrm{ref}}$ is the wavelength shift of the R_1 peak centre position under pressure.

The error propagation can be expressed using the general formula:

$$\sigma_P^2 = \left(\frac{\partial P}{\partial A}\sigma_A\right)^2 + \left(\frac{\partial P}{\partial B}\sigma_B\right)^2 + \left(\frac{\partial P}{\partial \lambda_{\text{sample}}}\sigma_{\text{sample}}\right)^2 + \left(\frac{\partial P}{\partial \lambda_{\text{ref}}}\sigma_{\text{ref}}\right)^2 \qquad (3)$$

Where the partial derivatives using $x = \frac{\Delta\lambda}{\lambda_{\text{ref}}}$ can be expressed as follows:

$$\frac{\partial P}{\partial A} = x \times 10^3 \times (1 + Bx),$$

$$\frac{\partial P}{\partial B} = A \times 10^3 \times x^2,$$

$$\frac{\partial P}{\partial \lambda_{\text{sample}}} = A \times 10^3 \times \left(\frac{1}{\lambda_{\text{ref}}}\right) \times (1 + 2Bx),$$

$$\frac{\partial P}{\partial \lambda_{\text{ref}}} = A \times 10^3 \times \left(\frac{-\lambda_{\text{sample}}}{\lambda_{\text{ref}}^2}\right)(1 + 2Bx).$$

The standard uncertainties in A and B were reported as $\sigma_A = 0.01$ and $\sigma_B = 0.03$ (Shen *et al.*, 2020). The uncertainties in λ_{sample} and λ_{ref} arise from a combination of instrumental uncertainty and the uncertainty in determining the peak center from spectral fitting and can be expressed as:

$$\sigma_{\lambda_i} = \sqrt{\sigma_{\text{instrument}}^2 + \sigma_{\text{fit}}^2} \qquad (4)$$

The general error propagation formula assumes all the variables are uncorrelated. However, if the samples and references are measured using the same spectrometer with a systematic calibration routine, the uncertainties in λ_{sample} and λ_{ref} can be correlated, which can be described with an additional term:

$$2 \times \frac{\partial P}{\partial \lambda_{\text{sample}}} \times \frac{\partial P}{\partial \lambda_{\text{ref}}} \times \rho \times \sigma_{\text{sample}} \times \sigma_{\text{ref}} \qquad (5)$$

where ρ is the correlation factor. In case of positively correlated measurements ($\rho > 0$) this causes the cancellation of errors, which indicates why it is useful to perform the measurements of samples and references on the same instrument with good calibration:

$$-2 \times A^2 \times 10^6 \times \left(\frac{\lambda_{\text{sample}}}{\lambda_{\text{ref}}^3}\right) \times (1 + 2Bx)^2 \times \rho \times \sigma_{\text{sample}} \times \sigma_{\text{ref}} \qquad (6)$$

The Error Propagation calculator uses the inputs to calculate the terms and final error propagation. With the use of a slider for the ρ correlation factor between λ_{sample} and λ_{ref}, it is possible to interactively observe the effect of error cancellation or amplification when the value is changed from no correlation ($\rho = 0$) to perfect positive ($\rho = 1$) or negative ($\rho = -1$) correlation, respectively.

S4. Gasket thickness calculator

The thickness *d* of the gasket in a diamond anvil cell can be calculated from the interference spectrum of white light based on the Fabry–Perot fringe patterns created when the incident beam is reflected multiple times between two sample-diamond interfaces (Kim *et al.*, 2021). The condition for constructive interference can be expressed as:

$$2nd = m_i \times \lambda_i = m_i \times \frac{1}{\bar{\nu}_i} \qquad (7)$$

where n is the refractive index of the medium, d is the gasket thickness, m is the interference order, and λ is the wavelength corresponding to the interference maximum, also written as wavenumber $\bar{\nu} = \lambda^{-1}$.

The distance between 2 interference fringes in the spectrum then becomes:

$$\bar{\nu}_i - \bar{\nu}_j = \Delta\bar{\nu} = \frac{\Delta \mathrm{m}}{2nd} \qquad (8)$$

where $\bar{\nu}_i$ and $\bar{\nu}_j$ are the wavenumbers of the intereference peak centers, and Δm is the difference in the interference order. If the gasket hole is empty (filled with air), then the equation can be simplified as:

$$\Delta\bar{\nu} = \frac{\Delta \mathrm{m}}{2d} \qquad (9)$$

When the interference spectrum is measured in cm^{-1} on the Raman spectrometer, the equation for the gasket thickness in μm can be expressed as:

$$d[\mu\mathrm{m}] = 10^4 \times \frac{\Delta m}{2\Delta\bar{\nu}} = 5 \times 10^3 \times \frac{\Delta m}{\bar{\nu}_i - \bar{\nu}_j} \qquad (10)$$

An interference spectrum (Figure S4) was recorded using the Horiba Jobin-Yvon LabRAM HR spectrometer, with the laser turned off and the diamond anvil cell (DAC) containing an air-filled gasket hole illuminated by white light. The experimental data were corrected using the BEADS baseline correction method. In the example shown, interference peaks at 915.11 cm^{-1} and 1149.18 cm^{-1}, corresponding to an interference order of 5, can be used in the Gasket Thickness calculator (Figure 3) to determine a gasket thickness of 106 μm.

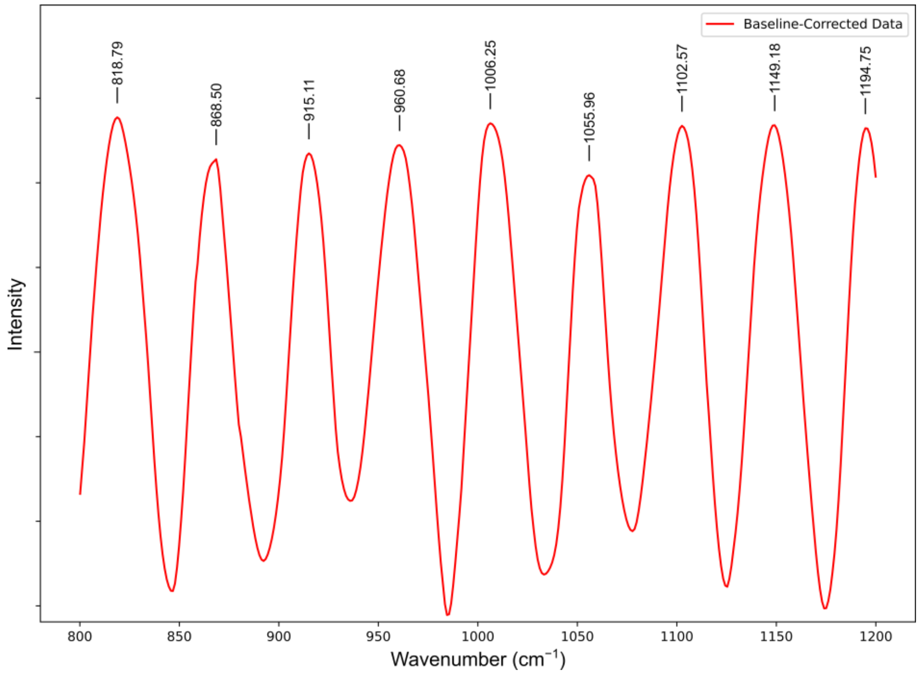

Figure S4 Baseline-corrected interference spectrum for gasket thickness measurement.
